# Retrospective Bayesian Reanalysis of Single Gentamicin Concentrations: A Neonatal Case Series

**DOI:** 10.3390/medicines12020009

**Published:** 2025-04-10

**Authors:** Staci L. Hemmer, Sarah K. Scoular

**Affiliations:** Skaggs School of Pharmacy, University of Montana, Missoula, MT 59801, USA; sarah.scoular@umontana.edu

**Keywords:** gentamicin, neonatal, Bayesian, pharmacokinetics, InsightRx^®^

## Abstract

**Background/Objectives**: Nomograms for adjusting gentamicin therapy in neonates using a single concentration are limited. The Dersch–Mills nomogram is inefficient for short-duration therapies, while the NeoFax nomogram is outdated based on the current American Academy of Pediatrics (AAP) guidelines. Bayesian software has shown accuracy for vancomycin in adults, but its performance for gentamicin in neonates is unclear. This study evaluates the accuracy of Bayesian estimation in predicting peak and trough gentamicin concentrations from a single measured level in neonates. **Methods**: A single-center, retrospective re-analysis was conducted of gentamicin concentrations in neonates. InsightRx^®^ was used to estimate maximum and minimum concentrations in a dosing interval (Cmax and Cmin) based on a single peak or trough concentration. Bias and accuracy were characterized using the mean difference (MD) between estimated and measured concentrations and the 95% limits of agreement (LOA) for the differences (±1.96 × SD). **Results**: Fifty-seven neonates (73 peak/trough pairs) were analyzed. Median gestational age was 34 weeks and median postnatal age was 0 days. The MD (LOA) between Cmin estimates and measured troughs was 0.03 mg/L (−0.17 to 0.13) for the trough-only analysis and 0.21 mg/L (−0.38 to 0.8) for the peak-only analysis. The MD (LOA) between Cmax estimates and measured peaks was 0.16 mg/L (−3.2 to 3.3) for the trough-only analysis and 1.2 mg/L (−0.58 to 3.0) for the peak-only analysis. **Conclusions**: In neonates, a Bayesian analysis of a trough concentration produces reliable Cmin estimates but is not as accurate in estimating Cmax. Analyzing a peak concentration produces Cmax and Cmin values that overestimate true concentrations. If the goal of monitoring is to ensure sufficiently low troughs, a single-level analysis is reasonable if levels are drawn near the end of the dosing interval, but Cmin predictions based on levels drawn early in the dosing interval should be avoided.

## 1. Introduction

Gentamicin and ampicillin are recommended as an empiric antimicrobial therapy for the treatment of neonatal sepsis [1,2]. In early-onset sepsis, antibiotics are discontinued after 48 h if blood cultures are negative and clinical signs of infection are absent. Therapeutic drug monitoring of gentamicin is recommended in neonates to maximize therapeutic efficacy and minimize the risk of nephrotoxicity and ototoxicity. Gentamicin peak concentrations best predict efficacy, while trough concentrations are correlated with toxicity [3]. The American Academy of Pediatrics (AAP) endorses gentamicin peak concentrations of from 8 to 12 mg/L, although a wider range of acceptable concentrations between 5 and 18 mg/L has been pursued [4,5,6,7]. The AAP also endorses trough concentrations of less than 2 mg/L, although many dosing nomograms strive to obtain gentamicin trough concentrations less than 1 mg/L [4,5,6,7,8].

Numerous strategies have been suggested to refine gentamicin dosing regimens from a single measured concentration, rather than from a pair of peak and trough concentrations. Adjusting the dosing interval for a 5 mg/kg dose based on a level measured 22 h after the first dose successfully produced trough concentrations of less than 2 mg/L (with approximately 80% less than 1 mg/L) and peak concentrations greater than 5 mg/L (with approximately 75% between 8 and 12 mg/L) [5,6]. A drawback of this nomogram is the requirement of an early, additional blood draw in neonates, the majority of whom discontinue the medication after one more dose. In addition, the initial dose of 5 mg/kg does not match AAP empiric dosing recommendations for most neonates [4]. An alternative nomogram using a random concentration after the first dose, conveniently collected through a clinical blood draw, has been investigated [8]. Unfortunately, the achievement of target troughs less than 1 mg/L could not be reliably predicted from the random level [8]. Other single-concentration dosing nomograms were developed from initial gentamicin dose regimens no longer endorsed by the American Academy of Pediatrics (AAP) [7,9].

An alternative strategy for the single-level monitoring of gentamicin involves Bayesian estimation of patient-specific pharmacokinetic parameters from a single concentration to predict gentamicin peak and trough concentrations. Compared to dosing nomograms, Bayesian estimation offers greater flexibility by allowing drug concentrations to be measured at any point during the dosing interval or throughout therapy (e.g., not at steady-state). Bayesian estimation also does not require specific fixed doses or consistent dosing intervals and may allow for therapeutic monitoring to take place with just a single concentration measurement. For vancomycin, evidence increasingly supports the use of a single concentration to estimate the vancomycin area-under-the-curve (AUC) in adults [10,11,12,13]. Traditionally, calculating the AUC required two concentrations: a peak and a trough. Evidence is limited regarding the efficacy of Bayesian dosing programs for estimating neonatal pharmacokinetics from a single concentration, whether for vancomycin or gentamicin. A retrospective simulation of gentamicin dosing in neonates using a Bayesian dosing program (InsightRx^®^) demonstrated improved target attainment compared to that predicted with a standard gentamicin dosing nomogram [14]. Almost half of the Bayesian estimates were based on a single trough. An important limitation was the use of Bayesian predictions of peak and trough concentrations to assess target attainment, rather than actual measured concentrations. Furthermore, since the publication of the study, InsightRx^®^ adjusted the population model to fix a systematic bias leading to overestimation of Cmin concentrations [15].

The purpose of this study was to evaluate the accuracy of estimates of gentamicin Cmax and Cmin concentrations using a Bayesian dosing program (InsightRx^®^, v1.44) and a single concentration (peak or trough). While single-level monitoring of gentamicin in neonates has been implemented using nomograms, little information is available to guide the single-level monitoring of gentamicin in neonates using Bayesian estimation. In particular, this evaluation aimed to characterize the reliability of concentration estimates at the opposite end of the dosing interval from the single measured peak or trough. Commercial Bayesian software programs will provide Cmax and Cmin estimates regardless of when the single level is measured. More information is needed to help clinicians properly interpret these estimates.

## 2. Materials and Methods

A single-center, retrospective reanalysis of gentamicin concentrations in neonates receiving intravenous gentamicin was conducted at a community hospital in Missoula, Montana with a level 3b neonatal intensive care unit (NICU). During the period of data collection from January 2018 to February 2021, neonates were dosed according to the NeoFax nomogram (Table 1) [9]. Per pharmacy dosing protocol, serum concentrations were ordered 30 min before the start of and 30 min after the completion of a dose administered between 4 and 4.5 days into therapy, depending on the dosing interval. Dose adjustments during this time period were based on first-order pharmacokinetic equations and commercial dosing software was not used.

Neonates in the NICU were eligible for inclusion if they received gentamicin therapy for more than three days with a peak and trough concentration drawn proximal to the same dose. Patients were excluded if the trough was drawn earlier than 120 min before the next scheduled dose or if the peak was drawn more than 90 min after the end of the infusion.

Gentamicin doses, serum creatinine concentrations, and weights were entered into a retrospective patient profile created in the Bayesian pharmacokinetics program (InsightRx^®^, v1.44). A single peak or trough concentration was analyzed using the original Fuchs model as the Bayesian prior [16]. The Fuchs model calculates the population clearance (CL) as a function of body weight (BW), gestational age (GA), and postnatal age (PNA) according to the following equation:

$$CL=0.089\times {\left(\frac{BW}{2170}\right)}^{0.75}\times \left[1+1.870\times \left(\frac{GA-34}{34}\right)\right]\times \left[1+0.054\times \left(\frac{PNA-1}{1}\right)\right]$$

where BW is measured in grams, GA is measured in weeks, and PNA is measured in days. For retrospective profiles in InsightRx^®^, PNA is calculated as the difference between the “First Dose Date” and the date of birth. For each retrospective record, the “First Dose Date” was entered as the day concentrations were measured to ensure that the PNA used in the clearance calculation matched the PNA at the time of the level assessment. The concentration was analyzed using the MAP Bayesian fitting method and a linear gradient weighting scheme. The steady-state maximum and minimum concentrations in a dosing interval estimated by the program, Cmax and Cmin, were recorded when only one concentration (peak or trough) was analyzed.

Serum gentamicin concentrations were determined by particle-enhanced turbidimetric inhibition immunoassay. Prior to April 2020, the QMS Gentamicin Assay (Thermo Fisher Scientific, Fremont, CA, USA) on a Roche-compatible analyzer was used. Starting in April 2020 a different assay was used, the Atellica CH Gentamicin Assay (Siemens Healthineers, Tarrytown, NY, USA) on the Atellica CH Analyzer. Consequently, the lower detection limit of the gentamicin serum concentration assay changed from 0.3 mg/L to 0.5 mg/L in April 2020. Concentrations below the lower limit of detection, reported as either <0.3 mg/L or <0.5 mg/L, were considered to be equal to 0.3 mg/L or 0.5 mg/L, respectively, in both the Bayesian program and in the statistical analysis.

Multiple peak/trough pairs measured in the same neonate were included. For levels measured subsequent to the initial peak/trough pair, only a single concentration was analyzed in the Bayesian program. Previous levels were not included in the fit in order to assess the accuracy of the Fuchs population in predicting concentrations at larger PNAs, without the influence of earlier concentrations.

Agreement between estimated Cmax/Cmin concentrations and measured peak/trough concentrations was assessed in the spring of 2023 after InsightRx^®^ corrected a systematic bias in their neonatal pharmacokinetic model [15]. The mean difference (MD) between the estimated concentrations and the measured concentrations was calculated to assess for bias. The 95% limits of agreement (LOA) denoted the accuracy of predictions, defined as MD ± 1.96 × SD. A Bland–Altman plot and line of best fit was used to display the differences and characterize proportional bias. The distribution of estimated Cmax concentrations and measured peak concentrations across the following designated ranges were compared with the McNemar–Bowker test: <8 mg/L, between 8 and 12 mg/L, and >12 mg/L. The distribution of estimated Cmin concentrations and measured trough concentrations above and below 1 mg/L were compared using the McNemar test. Excel 2021 was used to collect data and calculate averages and measures of variability. Statistical tests were performed using R Version 4.2.0. The Strengthening the Reporting of Observational Studies in Epidemiology (STROBE) checklist was used to ensure the transparent reporting of the study design, data collection, and analysis.

## 3. Results

Over the 3-year review period, 333 neonates received at least one dose of gentamicin, with 94 neonates receiving gentamicin for greater than 3 days. Of the 94 eligible neonates, 26 were excluded because a peak or trough concentration was not measured (22 neonates) or was not measured in the acceptable time window (4 neonates). Eleven neonates were not reviewed because of time constraints. Ultimately, 57 neonates met the inclusion and exclusion criteria, contributing a total of 73 peak/trough pairs. The majority of neonates started gentamicin therapy on the first day of life and received an initial regimen of 4 mg/kg every 24 h (Table 2). Thirteen (22.8%) started therapy at a postmenstrual age of less than 30 weeks. All but one neonate had a serum creatinine of less than 1 mg/dL. One neonate was also receiving intravenous dopamine therapy, which can decrease gentamicin clearance [16]. Therapeutic hypothermia as a treatment for hypoxic ischemic encephalopathy (HIE) also reduces gentamicin clearance, but no patient in this study was diagnosed with HIE [17].

**Figure 1:**
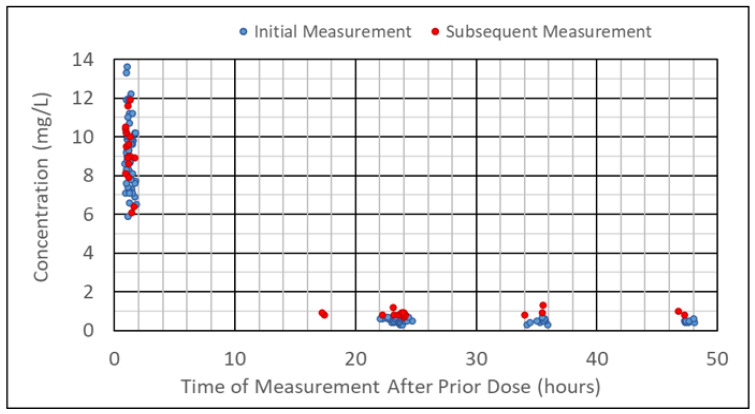
Time and magnitude of measured peak and trough concentrations relative to the prior dose. Subsequent measurements are additional peak/trough pairs measured in the same neonate after the initial set of peak/trough measurements.

The mean measured peak concentration was 9.14 mg/L (range 5.9–13.6 mg/L). The mean measured trough concentration was 0.61 mg/L (range 0.3–1.3 mg/L). The time of the measured concentrations after the previous dose is shown in Figure 1. Sixteen troughs were reported as undetectable and were rounded up to either 0.3 mg/L or 0.5 mg/L, depending on the assay used.

### 3.1. Estimated Cmax Compared to Measured Peak Concentrations

**Figure 2:**
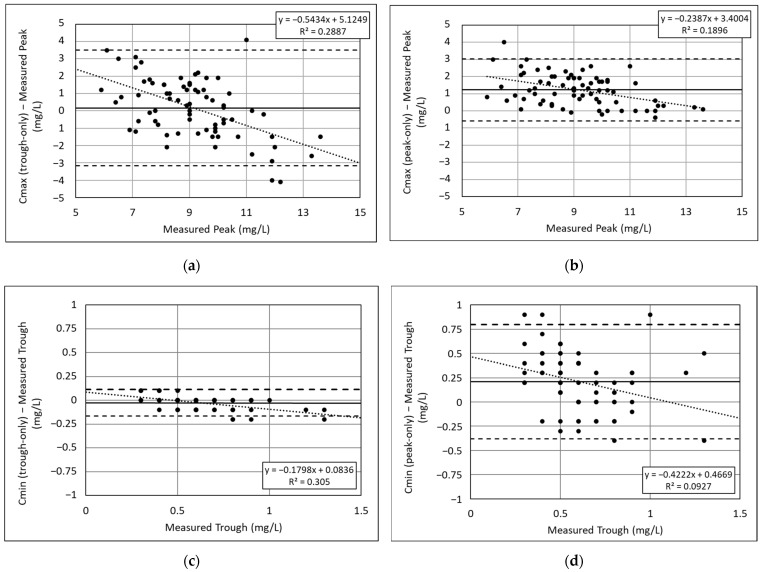
Bland–Altman plots showing the differences between: (**a**) trough-only estimate of Cmax and the measured peak concentration; (**b**) peak-only estimate of Cmax and the measured peak concentration; (**c**) trough-only estimate of Cmin and the measured trough concentration; (**d**) peak-only estimate of Cmin and measured trough concentration. Data points represent the difference of the estimated and measured concentrations. The solid line represents the mean difference between the estimated and measured concentrations. The dashed lines represent the 95% limits of agreement (LOA), determined by ±1.96 × SD of the differences.

With the trough-only analysis, the mean difference between the estimated Cmax and the measured peak concentration was 0.16 mg/L (Figure 2a). While the mean difference was small, a relatively large variability was observed with a 95% LOA of −3.2 to 3.3 mg/L. Furthermore, the difference between the estimated Cmax and measured peak concentrations displayed proportional bias, with the line of best fit decreasing by 0.54 mg/L with each 1 mg/L increase in the measured peak concentration. With the peak-only analysis, the mean difference between the estimated Cmax and the measured peak concentration was greater than the trough-only analysis at 1.2 mg/L (Figure 2b). However, the variability was lower with the peak-only analysis, with a 95% LOA of −0.58 to 3.0 mg/L. Less proportional bias was observed, with the line of best fit decreasing by 0.24 mg/L with each 1 mg/L increase in the measured peak concentration.

Regarding the agreement between the measured Cmax concentrations across designated categories, the Cmax estimates produced by the trough-only analysis were similarly distributed across the designated categories compared to the measured peaks (*p* = 0.21), although 16 pairs (22%) were discordant (Table 3). The distribution of the Cmax estimates produced by the peak-only analysis across the designated categories was significantly different from the distribution of measured peaks (*p* = 0.0002), with 17 (23%) discordant pairs.

### 3.2. Estimated Cmin Compared to Measured Trough Concentrations

With the trough-only analysis, the mean difference between the estimated Cmin and the measured trough concentration was −0.03 mg/L (Figure 2c). The LOA was −0.17 to 0.13 mg/L. Minimal proportional bias was observed, with the line of best fit decreasing by 0.18 mg/L with each 1 mg increase in the measured trough concentration. With the peak-only analysis, the mean difference between the estimated Cmin and the measured trough concentration was greater than the trough-only analysis at 0.21 mg/L (Figure 2d). The variability was also greater with the peak-only analysis, with a 95% LOA of −0.38 to 0.8 mg/L. More proportional bias was observed with the peak-only analysis, with the line of best fit decreasing by 0.42 mg/L with each 1 mg/L increase in the measured peak concentration, but the coefficient of determination was low (R^2^ =0.093).

Compared to the distribution of measured trough concentrations above and below 1 mg/L, the Cmin estimates produced by the trough-only analysis were identically distributed (*p* = 1), with no discordant pairs (Table 4). The peak-only analysis produced a Cmin distribution that was statistically different to the distribution of measured troughs (*p* = 0.0023). In fact, 12 out of the 70 patients (17%) with measured trough concentrations less than 1 mg/L would have an estimated Cmin greater than 1 mg/L with the peak-only analysis.

## 4. Discussion

The purpose of this study was to investigate the accuracy of a Bayesian software program (InsightRx^®^) in predicting neonatal gentamicin concentrations when analyzing a single peak or a single trough concentration. The results provide support for the use of the trough-only analysis to estimate Cmin concentrations, but the peak-only analysis does not reliably predict Cmin values. The trough-only analysis produced Cmin estimates that best matched the measured trough, with minimal bias and accuracy within 0.17 mg/L of the measured trough. In addition, the trough-only analysis produced Cmin estimates that were identically distributed above and below 1 mg/L. The Cmin estimates produced by the peak-only analysis had a larger bias and less accuracy, with potential differences up to 0.8 mg/L. Greater discordance was observed across designated categories ≤ 1 mg/L and >1 mg/L and unnecessary dose reductions would have occurred for 16% of the peak-only analyses if a Cmin value of less than 1 mg/L was desired.

It is not surprising that the trough-only analysis produced Cmin estimates that better match the measured trough concentrations. The Cmin concentrations were estimated at exactly at the end of the dosing interval, while the trough concentrations were drawn, on average, 30 min prior to the end of the interval. As long as the Bayesian fit produces a concentration versus time curve that reasonably matches the measured trough, an additional 30–60 min of exponential decay from the measured trough to the Cmin value is unlikely to produce much change. With the peak-only analysis, greater bias and less accuracy was observed in the Cmin estimates because 23 or more hours of exponential decay occurred between the measured peak concentration and the estimated Cmin. Therefore, the a priori population model used to fit the peak concentration had a much greater influence on the estimated Cmin concentrations. The positive bias observed with the peak-only analysis suggests that the Fuchs population model overestimates trough concentrations, possibly because the Fuchs model was built from a data set that did not include true troughs but instead was developed from peak levels and 18 h levels [16].

While Cmin estimates from the trough-only analysis agreed well with the measured trough concentrations, the estimates of Cmax demonstrated less agreement with the measured peaks, regardless of which concentration was analyzed. Either 17 or 18 estimates of Cmax were discordant with the measured peak across the clinical categories of <8 mg/L, 8–12 mg/L, and >12 mg/L when analyzing a single trough or a single peak. The trough-only analysis had less overall bias than peak-only analysis (0.16 mg/L versus 1.22 mg/L), but the estimates were less accurate, with expected absolute differences up to 3 mg/L. Furthermore, a proportional bias was seen with the trough-only estimates of Cmax, which more commonly overestimated measured peaks less than 9 mg/L and underestimated peaks greater than 9 mg/L. This observation suggests that, with standard empiric dosing, the Fuchs population model anticipates Cmax concentrations to be around 9 mg/L, and that the measured trough has only a minor influence on the estimated Cmax concentrations.

The observed bias between the peak-only Cmax estimates and the measured peaks is expected. The Cmax concentrations were estimated exactly at the end of the infusion, while peak concentrations were drawn, on average, 41 min after the end of the infusion. Consequently, the estimated Cmax concentration should be higher than the measured peak, especially because the Fuchs model is a two-compartment model with a distribution half-life within the order of one hour. No consensus exists regarding whether a back-calculated Cmax concentration should be used to assess peak target attainment, or if a peak concentration drawn 30 min after the end of the infusion is more appropriate [18]. Advocates for using a back-calculated Cmax to assess target attainment assumed the use of a one-compartment model, which would produce smaller estimates of Cmax than a two-compartment model with a one-hour distribution half-life, as was used by InsightRx^®^ [3,5].

This study used AAP suggested target concentrations of <8, 8–12, and >12 mg/L to assess the concordance between estimates of Cmax and measured peaks. Other single-concentration nomograms for dosing gentamicin in neonates adopted a wider range of acceptable peak values between 5 and 15 mg/L [5,6,7,9]. Using the wider range, the trough-only analysis would have produced one discordant pair (15.1 mg/L estimated vs. 11.0 mg/L measured) while the peak-only analysis would have resulted in 100% agreement. In this scenario, the trough-only analysis showed reasonable agreement between the estimated Cmax and Cmin concentrations and measured peaks and troughs.

Accepting a wider range of peak concentrations reduces the need for monitoring or estimating peak concentrations. For example, all peak concentrations in this study were in the expanded range of 5–15 mg/L with the empiric dosing used. Previous nomograms accepting this wider range of peaks instead focused on ensuring trough concentrations were below 1 mg/L [5,6,7,9]. If a single trough concentration is sufficient to monitor gentamicin in neonates, the added benefit of estimating Cmin concentrations from measured trough concentrations using Bayesian software may not be obvious. Because of the close agreement between Cmin estimates and the measured troughs observed in this study with the trough-only analysis, Bayesian estimation may allow for a wider time frame for measuring trough concentrations and extrapolation to Cmin values. For example, the Dersch–Mills nomogram measures the first level 22 h after the infusion, rather than 24 h, to allow for analysis and adjustment before the next scheduled dose [5,6]. Bayesian estimation software could also allow for earlier trough measurements, 2 h before the scheduled dose, and the assessment of extrapolated Cmin values for dose adjustment. Measuring an earlier trough would also reduce the number of undetectable levels. Undetectable levels can cause clinical unease, as the time below the minimum inhibitory concentration (MIC) is unknown.

This study has several limitations. Only 13 patients, contributing 17 peak/trough pairs, had a post-menstrual age less than 30 weeks. More data are needed to characterize the accuracy of Bayesian estimation in neonates with a greater degree of prematurity. Only one patient had an acute kidney injury (AKI), with an increase in SCr of greater than 0.5 mg/L, so the accuracy of Bayesian estimation in this population is unknown. However, neither SCr nor urine output are covariates in the Fuchs model, so Bayesian estimation using this model would not be recommended in neonates with AKI [16]. Finally, InsightRx^®^ refined the Fuchs model after these data were collected, which improved the accuracy of the Cmax estimates (defined as differing by less than 2 mg/L), although two concentrations were used to make the estimates [19].

## 5. Conclusions

The accuracy of Bayesian analysis in predicting trough target achievement in neonates based on a single gentamicin concentration depends on the timing of the measurement. Concentrations measured early in the dosing interval tend to produce overestimates of the true trough (Cmin) and are therefore unreliable for predicting target attainment. In contrast, concentrations drawn closer to the end of the dosing interval provide a more accurate prediction. Bayesian techniques may help expand the acceptable time window for measuring trough concentrations while maintaining reliable predictions.

## Figures and Tables

**Table 1:** Initial gentamicin dosing and monitoring protocol for neonates.

PMA (Weeks)	PNA (Days)	Dose (mg/kg)	Interval (Hours)	Draw Concentrations Proximal to:
≤29	0 to 7	5	48	3rd dose
	8 to 28	4	36	4th dose
30 to 34	0 to 7	4.5	36	4th dose
	≥8	4	24	5th dose
≥35	ALL	4	24	5th dose

PMA = postmenstrual age as defined by gestational age + postnatal age; PNA = postnatal age.

**Table 2:** Demographic and dosing details for neonates receiving gentamicin therapy.

Characteristic	Value	Unit
Mean Gestational Age (SD)	34.3 (5.0)	weeks
Median Postnatal Age (IQR)	0 (0–2)	days
Median Postmenstrual Age (IQR)	35.4 (29.6–37.9)	weeks
Mean Weight (SD)	2.34 (0.97)	kg
Mean Serum Creatinine (SD)	0.41 (0.19)	mg/dL
Initial Dosing Regimen		
5 mg/kg every 48 h	9 (15.8%)	
4 mg/kg every 36 h	3 (5.2%)	
4.5 mg/kg every 36 h	7 (12.3%)	
4 mg/kg every 24 h	38 (66.7%)	
Median Draw Time (IQR)	4.0 (3.96–5.99)	days
Median Peak Time After End of Infusion (IQR)	41 (34–55)	minutes
Median Trough Time Before Next Dose (IQR)	30 (16.5–47)	minutes
Gentamicin Indication ^†^		
Sepsis	30 (52.6%)	
Pneumonia	10 (17.5%)	
Necrotizing Enterocolitis	7 (12.3%)	
Meningitis	5 (8.8%)	
Bacteremia	4 (7%)	
Tracheitis	2 (3.5%)	
Duodenal Atresia	1 (1.8%)	
Urinary Tract Infection	1 (1.8%)	
Omphalitis	1 (1.8%)	
Factors Affecting Gentamicin Pharmacokinetics		
Dopamine Co-Administration	1 (1.8%)	
HIE Requiring Therapeutic Hypothermia	0 (0%)	

^†^ Four patients had two listed indications for gentamicin therapy. HIE = Hypoxic Ischemic Encephalopathy.

**Table 3:** Distribution of measured peak concentrations and estimated Cmax concentrations across designated categories using a trough-only or peak-only analysis.

		Measured Peak			*p*-Value
		<8 mg/L	8–12 mg/L	>12 mg/L	
Estimated Cmax (trough-only fit)	<8 mg/L	10	5	0	*p* = 0.48
	8–12 mg/L	9	45	2	
	>12 mg/L	0	1	1	
Estimated Cmax (peak-only fit)	<8 mg/L	6	0	0	*p* = 0.0001
	8–12 mg/L	13	46	0	
	>12 mg/L	0	5	3	

**Table 4:** Distribution of measured trough concentrations and estimated Cmin concentrations across designated categories using a trough-only or peak-only analysis.

		Measured Trough		*p*-Value
		≤1 mg/L	>1 mg/L	
Estimated Cmin (trough-only fit)	≤1 mg/L	70	0	*p* = 1
	>1 mg/L	0	3	
Estimated Cmin (peak-only fit)	≤1 mg/L	58	1	*p* = 0.0023
	>1 mg/L	12	2	

## Data Availability

The data presented in this study are available on request from the corresponding author in order to maximize the privacy of patients included in the study.
